# Case Report: **“**Damage control” in obstetric neurosurgery: staged management of ruptured arteriovenous malformation with herniation

**DOI:** 10.3389/fsurg.2026.1845552

**Published:** 2026-05-08

**Authors:** Qunlong Jiang, Xiaoli Liu, Zhiwei Zhang, Xiaokui Kang

**Affiliations:** 1 Department of Neurosurgery, Liaocheng People’s Hospital, Liaocheng, China; 2 Department of Obstetrics and Gynaecology, Liao Cheng People’s Hospital, Liaocheng, China

**Keywords:** arteriovenous malformation, case report, damage control, normal perfusion pressure breakthrough (NPPB), pregnancy

## Abstract

**Aim:**

Catastrophic arteriovenous malformation (AVM) rupture during the second trimester presents a formidable clinical dilemma, necessitating a delicate balance between maternal resuscitation and fetal preservation. This challenge is acute when uncal herniation precludes extensive diagnostic workup.

**Material and methods:**

We report a staged “damage control” strategy in a 27-year-old gravida (26 weeks) who presented with acute neurologic collapse, anisocoria, and coma. In advance of definitive angiographic characterization, we proceeded directly to emergency decompressive craniectomy to reverse impending herniation. Postpartum angiography revealed a small, eloquent Spetzler–Martin Grade II AVM. Definitive embolization (Onyx-18) was deferred until two months postpartum to ensure hemodynamic stability.

**Results:**

The emergency decompression successfully stabilized the patient. Following 12 weeks of supportive care, the pregnancy culminated in a successful term delivery. The patient achieved a full neurological recovery (modified Rankin Scale (mRS) score of 0), and the infant remained healthy.

**Conclusion:**

This case substantiates that in the context of pregnancy complicated by herniation, neurosurgical intervention must decouple acute decompression from vascular treatment. A staged approach may facilitate maternal neurological recovery while preserving fetal viability.

## 1 Introduction

Rupture events of AVM are particularly catastrophic in the maternal population, accounting for 5%–12% of maternal deaths and carrying case fatality rates approaching 83% (1). The “damage control” philosophy originated in trauma surgery, prioritizing immediate survival over a complete anatomical cure. In obstetric neurosurgery, we apply this concept by separating the emergency brain decompression from the high-risk vascular treatment. This staged approach minimizes immediate surgical stress on both the mother and the fetus.

Here, we report the case of a pregnant woman at 26 weeks of gestation who presented with ruptured AVM complicated by acute subdural hematoma and herniation. We first describe the patient's severe initial symptoms, physical examination results and emergency neuroimaging features. On this basis, we explain why immediate decompression should be performed prior to etiological diagnosis. We also clarify that stable maternal condition is the essential premise for fetal survival. Such a strategy, however, remains controversial and is seldom detailed in the literature, particularly in the context of advanced pregnancy.

## 2 Case description

The timeline of the patient's episode of care is summarized in Table 1. Following the initial emergency decompressive craniectomy (DC) on Day 1, the pregnancy was successfully maintained until a planned cesarean delivery at 39 weeks of gestation (93 days post-DC). Postpartum diagnostic DSA and definitive Onyx embolization were subsequently performed 76 days after delivery. Finally, cranioplasty was successfully completed 75 days post-embolization, making the conclusion of the surgical interventions with an excellent functional recovery.

**Table 1:** Timeline of clinical events, interventions, and outcomes.

Date/Time	Clinical event & intervention	Outcome/Status
September 4, 2024	Sudden collapse, GCS 6, anisocoria. CT showed massive ASDH. Emergency decompressive craniectomy (DC) performed.	Herniation reversed. Patient stabilized in ICU with strict MAP/ICP control.
December 6, 2024	Cesarean section performed under general anesthesia at 39 weeks gestation.	Delivery of a healthy infant. Postpartum MRI suggested a vascular lesion.
February 20, 2025	Readmission for definitive diagnostic workup.	DSA confirmed Spetzler-Martin Grade II AVM in the right frontoparietal lobe.
February 24, 2025	Targeted endovascular embolization with Onyx-18.	>95% occlusion achieved. Minimal residual left intentionally to prevent NPPB.
May 10, 2025	Cranioplasty performed using a custom-molded implant.	Uncomplicated surgical recovery.
6-Month Follow-up	Outpatient clinical assessment.	Patient is neurologically intact (GCS 15, left limb power 5/5). Infant shows normal development.

### 2.1 Clinical presentation and emergency management

**Figure 1:**
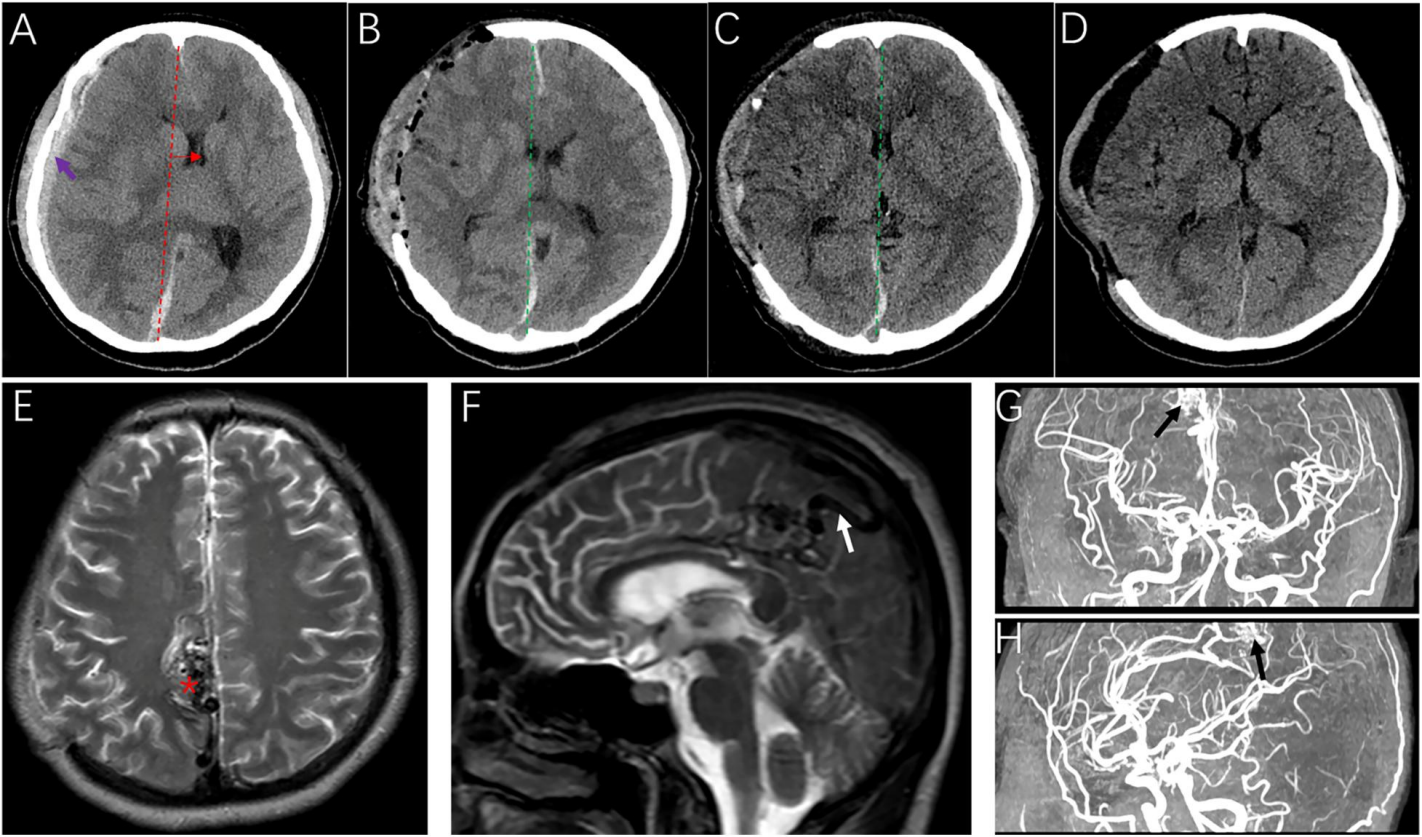
Radiologic evolution demonstrating the staged “damage control” management of a ruptured AVM in pregnancy. **(A)** Preoperative noncontrast computed tomography (CT) revealing a massive right-sided acute subdural hematoma (ASDH) with severe mass effect (purple arrow), effacement of the right lateral ventricle, and significant midline shift (the red dashed line and the red horizontal arrow), indicating impending uncal herniation. **(B,C)** Early postoperative CT scans showing the evacuation of the hematoma and the progressive restoration of the midline (green dashed lines) following emergent right decompressive craniectomy **(D)** Postoperative day 14 CT showing resolution of the brain swelling without midline shift. **(E,F)** Postpartum magnetic resonance imaging (MRI) (T2-weighted sequences) demonstrating an abnormal vascular signal (red asterisk) in the right frontoparietal lobe with a prominently dilated cortical draining vein (white arrow). **(G,H)** MRA confirming the presence of the arteriovenous malformation nidus (black arrows).

A 27-year-old female, gravida 2 para 1, presented to the Emergency Department on September 4, 2024, with a 2-hour history of thunderclap headache, nausea, and rapid deterioration of consciousness, followed by generalized tonic–clonic seizures. On admission, the Glasgow Coma Scale (GCS) score was 6 (E1V1M4). Pupils were anisocoric (right 5.0 mm, fixed; left 3.0 mm, reactive), consistent with uncal herniation. Urgent noncontrast CT revealed a significant acute subdural hematoma (SDH) with extension into the cerebral longitudinal fissure, causing a severe midline shift (Figure 1A). Given the imminent herniation, the patient was taken immediately to the operating room for a large right-sided decompressive craniectomy and hematoma evacuation.

### 2.2 Postoperative course and obstetrical care

Immediately postoperatively, the pupils returned to near-equal size. The patient was managed in the intensive care unit (ICU) with sedation and mechanical ventilation. Management focused on a multidisciplinary protocol balancing maternal mean arterial pressure (MAP) to ensure placental perfusion while strictly controlling intracranial pressure (ICP). Daily bedside fetal ultrasound confirmed fetal viability. After the patient achieved relative hemodynamic stabilization, cerebral Magnetic resonance angiography (MRA) was recommended to define the bleeding etiology and prevent recurrent hemorrhage. Nevertheless, the patient's family declined this diagnostic suggestion. Upon discharge from the initial hospitalization, she was conscious of left-sided hemiparesis (muscle strength: Grade 4/5).

### 2.3 Delivery and diagnostic workup

On December 6, 2024 (39 weeks gestation), a healthy infant was delivered via cesarean section under general anesthesia to avoid Valsalva maneuvers. During this admission, a noncontrast magnetic resonance image (MRI) suggested a vascular lesion in the right frontoparietal lobe (Figures 1E–H). Although digital subtraction angiography (DSA) was recommended, the family declined invasive investigation at that time, opting for discharge and outpatient follow-up.

### 2.4 Angiography evaluation and endovascular intervention

**Figure 2:**
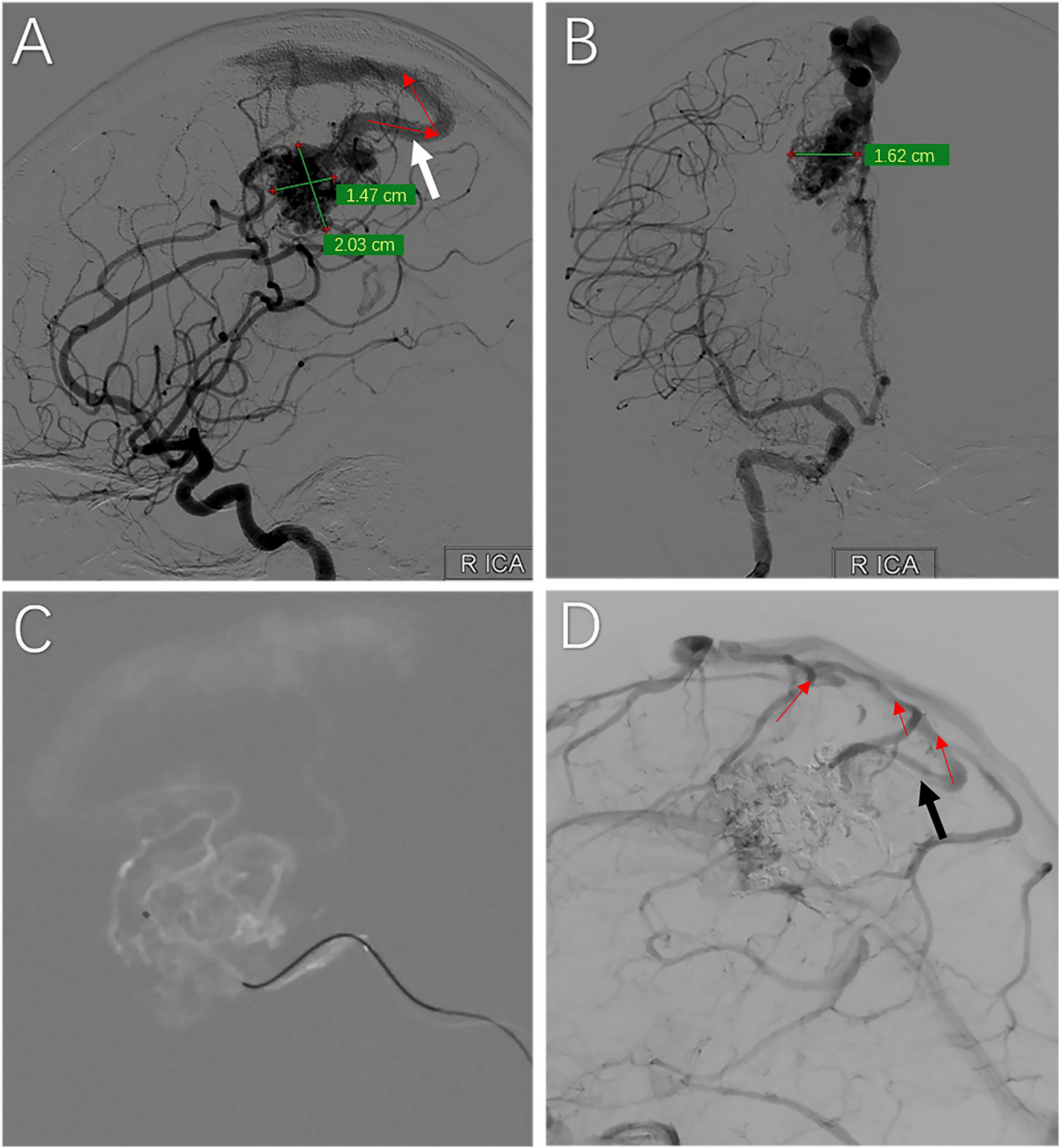
Diagnostic cerebral angiography and targeted endovascular embolization. **(A,B)** Pre-embolization digital subtraction angiography (DSA) revealing a Spetzler–Martin Grade Ⅱ arteriovenous malformation (AVM) nidus. The arterial supply is derived from the callosomarginal and pericallosal branches of the anterior cerebral artery (ACA). The large white arrow indicates the origin of the robust superficial draining vein, while the series of red arrowheads demonstrate its flow direction emptying into the superior sagittal sinus.**(C)** Intraoperative angiogram demonstrating super-selective microcatheter navigation into the distal feeding pedicles of the right callosomarginal and pericallosal arteries prior to Onyx-18 injection. **(D)** Final control angiogram following staged embolization. The image demonstrates near-complete occlusion (>95%) of the nidus with strict preservation of the main venous outflow (black arrow) and other venous drainage pathway (red arrowheads).

On February 20, 2025 (2 months post-postpartum), the patient was readmitted for definitive management. DSA revealed a nidus measuring approximately 20.3mm × 14.7 mm × 16.2 mm in the right frontoparietal lobe (Figures 2A,B), involving the motor cortex. The arterial supply was derived from the pericallosal branch of the anterior cerebral artery (ACA), and venous drainage was superficial via dilated cortical veins into the superior sagittal sinus (SSS). The lesion was classified as Spetzler–Martin Grade II (size < 3 cm: 1 point; eloquence/motor cortex: 1 point; superficial drainage: 0 points).

On February 24, 2025, endovascular embolization was performed under general anesthesia. A microcatheter was navigated to the feeding pedicles (Figure 2C), and a total of 4.6 mL of Onyx-18 liquid embolic agent was injected in four distinct, prolonged stages to ensure nidus penetration while preserving venous outflow. Final control angiography demonstrated near-complete occlusion (> 95%) with a significant reduction in shunt flow. To minimize the risk of normal perfusion pressure breakthrough (NPPB), given the patient's history of rupture, the procedure was voluntarily terminated with a minimal residual nidus (Figure 2D). Strict systolic blood pressure control (< 120 mmHg) was maintained for 48 h post-operatively.

### 2.5 Postoperative follow-up and outcome

Cranioplasty was performed on May 10, 2025, using a custom-molded implant. At the final 6-month follow-up, the patient was neurologically intact (GCS 15, left limb power 5/5) with no seizures. The infant showed normal developmental milestones.

## 3 Discussion

**Figure 3:**
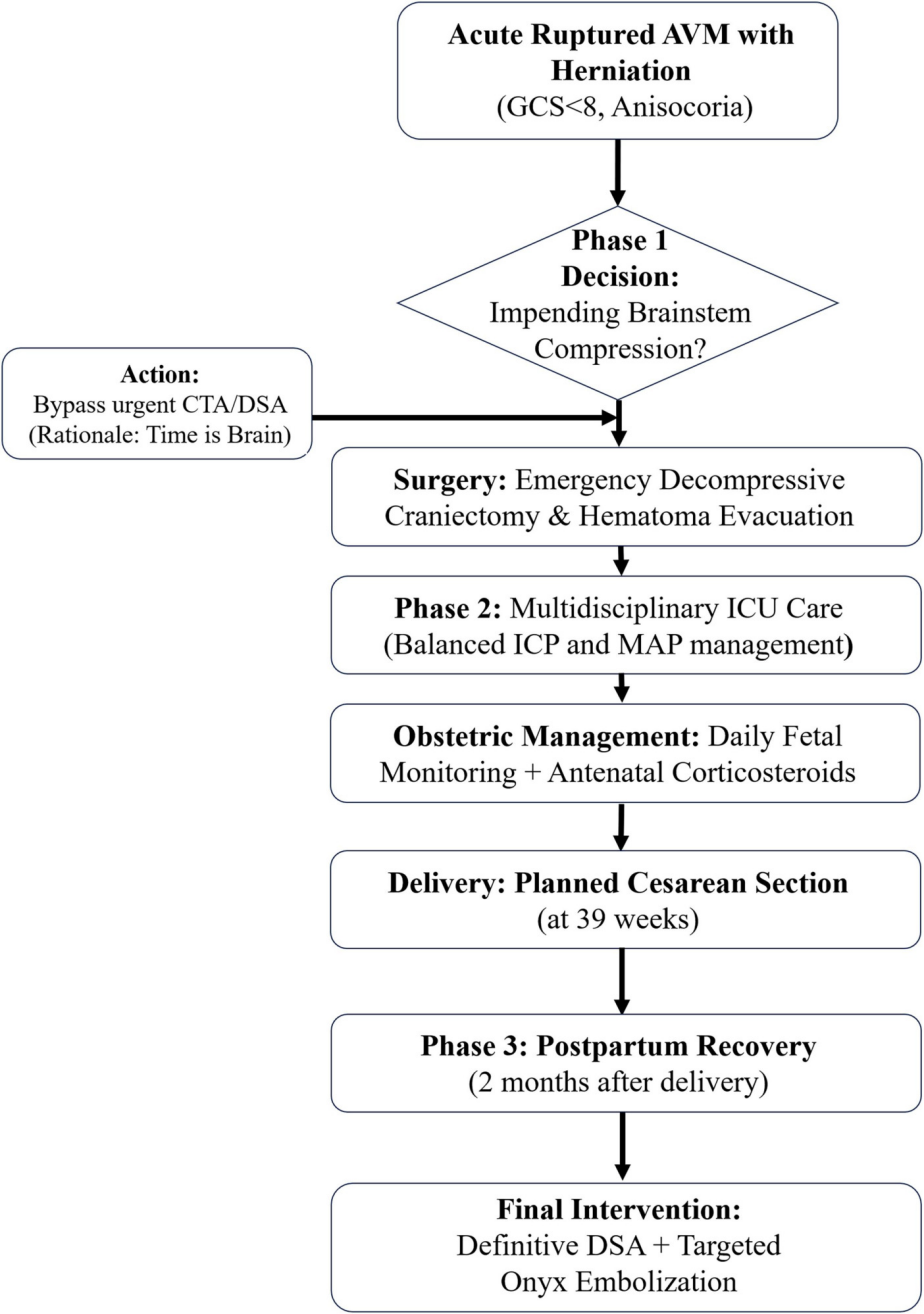
Clinical decision-making schematic for the staged "damage control” strategy in obstetric neurosurgery. This flowchart illustrates the strategic uncoupling of emergency mechanical decompression from definitive vascular treatment in a pregnant patient with ruptured AVM and impending herniation. In the hyperacute phase, life-saving decompressive craniectomy is prioritized by bypassing time-consuming vascular imaging once clinical signs of brainstem compression are identified. This is followed by a multidisciplinary maintenance phase to sustain the pregnancy until term delivery, and a final postpartum phase for definitive angiographic characterization and targeted endovascular embolization after the normalization of maternal hemodynamics.

Catastrophic intracranial hemorrhage with cerebral herniation during pregnancy is rare and is associated with substantial maternal and fetal mortality (2, 3). This case illustrates that when maternal survival—and by extension, fetal viability—is threatened by cerebral herniation, a strictly staged “damage control” protocol offers a favorable risk-benefit profile. The clinical decision-making and staged management schematic is presented in Figure 3.

### 3.1 The “damage control” imperative in obstetric neurosurgery

The crux of this case lies in the controversial decision to bypass vascular imaging in favor of immediate mechanical decompression. Although “blind” hematoma evacuation is associated with a theoretical risk of intraoperative aneurysmal or nidal rupture, this risk is probabilistic and surgically manageable. In contrast, mechanical brainstem compression imposes an immediate, deterministic outcome of irreversible ischemic injury and respiratory arrest. As articulated in the 2022 AHA/ASA Guidelines (4), the reversal of brainstem compression takes precedence over etiologic diagnosis. In the face of active herniation, the time cost of angiography is not merely a logistical delay; it is a direct contributor to irreversible brainstem infarction.

Ethical challenges in this case centered on the maternal-fetal conflict during emergency decision. When the mother is incapacitated, clinicians must navigate shared decision-making with the family, balancing the immediate need for maternal life-saving decompression against potential fetal distress. In this case, given the mother's critical condition, supportive care and fetal maturation were prioritized. Our strategy effectively uncouples intracranial pressure management from definitive vascular treatment. By converting a hyperacute crisis into a manageable chronic condition, we stabilized maternal physiology sufficiently to sustain the pregnancy. This approach adheres to the obstetrical axiom that maternal perfusion is the absolute prerequisite for fetal survival (5).

### 3.2 AVM characteristics and treatment strategy

With respect to the timing of definitive treatment, the management of intracranial AVMs during pregnancy remains a subject of intense debate. While some authors advocate for early intervention to prevent rerupture (6), others recommend deferring treatment until the postpartum period if the patient is stable (7, 8). Many literature reviews and reports underscore the complexity of the decision, and the core decision-making process hinging on balancing perioperative iatrogenic risks against the exceptionally high risk of rebleeding in the natural history of the disease (9–12). The intrapartum intervention is often advocated for ruptured AVMs to rapidly eliminate the exceedingly high risk of rebleeding, thereby mitigating the associated high mortality and morbidity rates. Current literature suggests that for pregnant patients at a gestational age of less than 34 weeks, early intervention within 2 weeks of the initial hemorrhage can effectively cut off the latent rebleeding crisis during the waiting period (10). However, performing invasive procedures during the acute phase means the patient and the fetus must confront additional surgical risks, including exposure to contrast media, ionizing radiation, anesthetic agents, and acute cerebral edema (13). Conversely, the advantage of postpartum intervention—the strategy adopted in our case—lies in avoiding the acute phase of hemodynamic instability, allowing the patient' physiological parameters to return to baseline before intervention to reduce surgical complications. As supported by recent literature and neurovascular physiology, deferring definitive endovascular embolization to the postpartum period avoids fetal exposure to ionizing radiation and embolic solvents (e.g., Onyx) (11). Furthermore, it allows maternal cardiac output to return to physiological baseline from its 40−50% gestational increase (14, 15). Hemodynamically, this staged approach avoids occluding high-flow shunts during a state of hypervolemia and vasoparalysis, theoretically minimizing the risk of NPPB (16, 17). Nevertheless, the major clinical pitfall of postpartum intervention is that the patient remains continuously exposed to a significantly elevated risk of rebleeding throughout the conservative waiting period (10). Extensive retrospective studies indicate that the rebleeding rate for AVMs with a history of prior hemorrhage is far greater than what is expected in their natural history, and there have been multiple reports of catastrophic rebleeding events occurring during conservative management (10, 18).

Intrapartum and postpartum interventions are not mutually exclusive, and clinical practice should not be rigidly confined to a single algorithm. An ideal decision-making paradigm should rely on a multidisciplinary, individualized assessment based on the angioarchitectural characteristics of the AVM, rupture status, and systemic hemodynamic vulnerability, ultimately seeking the optimal balance between “arresting immediate lethal risks” and "maintaining physiological stability" (10, 17).In this case, the interval between emergency decompression and a definitive diagnosis was prolonged because the family initially refused radiographic investigation during pregnancy. Retrospectively, postpartum DSA provided an angiographic explanation for the patient's stability during this high-risk interval. Imaging revealed a robust, dilated cortical vein with unimpeded outflow to the superior sagittal sinus. Unlike lesions with venous stenosis, which carry a high risk of imminent rerupture (18, 19), this favorable venous configuration likely facilitated efficient pressure dissipation. These findings offer critical pathophysiological insight: While the delay in treatment was clinically enforced rather than electively planned, the patient's favorable angioarchitecture (Spetzler–-Martin Grade II with superficial drainage) was the likely safeguard that prevented rebleeding despite the physiological stress of pregnancy.

### 3.3 Efficacy of embolization and NPPB prevention

Angiographic obliteration is the standard therapeutic goal for AVMs. However, high-flow vascular lesions require a nuanced management in patients with a history of hemodynamic instability. In this case, we achieved near-complete occlusion (>95%) using a staged injection of 4.6 mL of Onyx-18. The decision to voluntarily halt the procedure with a minimal residual nidus (<5%) was a deliberate clinical judgment rather than a technical limitation. Abrupt, complete occlusion of a high-flow shunt—particularly in a brain primed by prior herniation and pregnancy-associated hypervolemia—causes a substantial risk of NPPB (6, 16, 17). Consequently, our decision to leave a minimal residual nidus was not a technical concession but a deliberate prophylactic measure. We accepted a near-complete angiographic result and prioritized physiological safety over optimal radiological appearances. This principle guides the management of complex vascular lesions, whose core target is hemorrhage prevention instead of perfect imaging presentation. Thus, staged embolization with intentional subtotal occlusion represents an extension of the same damage-control philosophy applied in the surgical phase.

### 3.4 Limitations

The primary limitation of this case report was the absence of vascular imaging during the acute phase, stemming from the family's refusal of diagnostic workup. Consequently, the safety of the observational interval was retrospectively confirmed rather than prospectively assured. Ideally, noninvasive imaging should be performed once the patient is stabilized to rule out high-risk angioarchitectural features (e.g., intranidal aneurysms) that might mandate early intervention regardless of gestational status.

## 4 Conclusion

Ruptured AVMs presenting as acute subdural hematomas during pregnancy represent a neurosurgical crisis associated with prohibitive mortality. This case study validates a “damage control” paradigm: In the setting of active herniation, acute decompression must be decoupled from definitive vascular isolation. By deferring the treatment of the nidus until the postpartum period, this staged approach may allow for the normalization of maternal hemodynamics, thereby minimizing the risk of intraoperative rupture and NPPB. While limited by its single-case design, this report substantiates that a staged “damage control” approach serves as a hypothesis—generating paradigm for managing ruptured AVMs with herniation in pregnancy. Further registry-based studies are required to validate the safety and generalizability of this strategy.

## 5 Patient perspective

My family and I went through the most terrifying experience of our lives when I suddenly collapsed during my pregnancy. Because I was in a coma, my family had to make an agonizing decision, but they trusted the surgical team to save my life first. Waking up in the ICU and learning that I had survived brain surgery while my baby was still safe was a miracle. The decision to delay the treatment of the blood vessel malformation until after my delivery gave me peace of mind to focus on by baby's birth. Today, holding my healthy child and having fully recovered my physical abilities, we are profoundly grateful for the staged treatment strategy that saved both of our lives.

## Data Availability

The original contributions presented in the study are included in the article/Supplementary Material, further inquiries can be directed to the corresponding authors.
